# Differential associations between diet and prediabetes or diabetes in the KORA FF4 study

**DOI:** 10.1017/jns.2018.25

**Published:** 2018-12-27

**Authors:** Taylor A. Breuninger, Anna Riedl, Nina Wawro, Wolfgang Rathmann, Konstantin Strauch, Anne Quante, Annette Peters, Barbara Thorand, Christa Meisinger, Jakob Linseisen

**Affiliations:** 1 Helmholtz Zentrum München, German Research Center for Environmental Health (GmbH), Independent Research Unit Clinical Epidemiology, Ingolstädter Landstr. 1, 85764 Neuherberg, Germany; 2 Helmholtz Zentrum München, German Research Center for Environmental Health (GmbH), Institute of Epidemiology II, Ingolstädter Landstr. 1, 85764 Neuherberg, Germany; 3 German Center for Diabetes Research (DZD), München-Neuherberg, Ingolstädter Landstr. 1, 85764 Neuherberg, Germany; 4 Ludwig-Maximilians-Universität München, Chair of Epidemiology, UNIKA-T Augsburg, Neusässer Str. 47, 86156 Augsburg, Germany; 5 Deutsches Diabetes-Zentrum (DDZ), Institute for Biometrics and Epidemiology, Auf'm Hennekamp 65, 40225 Düsseldorf, Germany; 6 Helmholtz Zentrum München, German Research Center for Environmental Health (GmbH), Institute of Genetic Epidemiology, Ingolstädter Landstr. 1, 85764 Neuherberg, Germany; 7 Ludwig-Maximilians-Universität München, Chair of Genetic Epidemiology, IBE, Faculty of Medicine, 81377 Munich, Germany; 8 ZIEL – Institute for Food and Health, Technical University of Munich, Weihenstephaner Berg 1, 85354 Freising, Germany

**Keywords:** Alcohol, Coffee, Fruit, Meat: *enable*-Cluster, 24HFL, 24-h food list, BVSII, Bavarian Food Consumption Survey II, KORA, Cooperative Health Research in the Region of Augsburg, NGT, normal glucose tolerance, SSB, sugar-sweetened beverages, T2DM, type 2 diabetes mellitus, UDM, undetected diabetes mellitus

## Abstract

Type 2 diabetes mellitus (T2DM) is a global public health epidemic. Diet and lifestyle changes have been demonstrated as effective measures in managing T2DM and preventing or delaying the progression from prediabetes to diabetes, yet the relationship between diet, prediabetes and diabetes is still not entirely clear. The present study aimed to further elucidate the relationship between diet, diabetes and especially prediabetes. A total of 1542 participants of the cross-sectional, population-based Cooperative Health Research in the Region of Augsburg (KORA) FF4 study (2013/2014) were included in this analysis. Dietary intake was derived using a method combining information from a FFQ and repeated 24-h food lists. Glucose tolerance status was assessed via oral glucose tolerance tests in all participants without a previous physician-confirmed diagnosis of T2DM, and was classified according to the 2003 American Diabetes Association criteria. Crude and fully adjusted multinomial logistic regression models were fitted to examine associations between diet and prediabetes, undetected diabetes mellitus (UDM) and prevalent T2DM. After adjusting for major covariates, fruit was significantly inversely and total meat, processed meat, sugar-sweetened beverages and moderate alcohol significantly associated with UDM and/or prevalent diabetes. Sex-specific analyses showed that in men, coffee was significantly inversely (OR 0·80; 95 % CI 0·67, 0·96) and heavy alcohol significantly (OR 1·84; 95 % CI 1·14, 2·95) associated with prediabetes. Our findings on diet and T2DM are consistent with current literature, while our results regarding coffee, heavy alcohol consumption and prediabetes highlight new possible targets for primary prevention of the derangement of glucose homeostasis.

Type 2 diabetes mellitus (T2DM) is a major public health concern that places a significant burden on both the healthcare system and the individual. Costs associated with T2DM were estimated to be €33·3 billion in Germany in 2010, with total healthcare costs for people with diabetes estimated to be three times higher than for people without diabetes^(^^1^^)^. This disease is associated with numerous adverse outcomes and complications for the patient, especially concerning macro- and microvascular disease progression^(^^2^^,^^3^^)^. Prediabetes, the presence of blood glucose levels that are elevated above normal but are still below the threshold for T2DM, has been recognised as placing an individual at increased risk of developing T2DM^(^^4^^)^. What is more, some evidence suggests that the state of prediabetes itself is not benign and may already be associated with complications such as increased CVD risk, retinopathy, neuropathy and nephropathy^(^^5^^)^. It is estimated that between 18 and 24 % of the German population has prediabetes, and that 5–10 % of people with prediabetes may progress to diabetes each year without intervention^(^^6^^,^^7^^)^.

Lifestyle alterations, including dietary changes, offer an estimated 40–70 % reduction in the relative risk of the progression from prediabetes to diabetes^(^^6^^)^. Therefore, it is imperative to have a complete understanding of the impact that diet has on the risk of both prediabetes and diabetes in order to make effective dietary recommendations. While extensive, though not conclusive, research has been done on diet and the risk of diabetes, relatively little research has investigated the relationship between dietary intake and the risk of prediabetes^(^^8^^–^^12^^)^. Therefore, the aim of the present study was to examine the cross-sectional association between dietary intake, prediabetes and T2DM in the Cooperative Health Research in the Region of Augsburg (KORA) FF4 study population, in order to better inform future prospective and experimental investigations.

## Methods

### Study population

The KORA FF4 study (2013/2014) is the second follow-up examination of the population-based KORA S4 health survey, conducted in the city of Augsburg and two surrounding counties in Southern Germany between 1999 and 2001. Of the 4261 individuals included in the initial survey, 2279 individuals also participated in the KORA FF4 study. Details regarding the participation rate for KORA FF4 have been published previously^(^^13^^)^. Of the total 2279 individuals who participated in FF4, those with type 1 diabetes mellitus (*n* 6), unknown glucose tolerance status (*n* 93) or missing dietary intake data (*n* 638) were excluded from this analysis. There were complete covariate data for all remaining 1542 participants. A sensitivity analysis comparing FF4 participants who were included *v.* excluded from the present analysis showed few differences, though participants who were excluded had a higher prevalence of smoking and were less active (data not shown). This study was conducted according to the guidelines laid down in the Declaration of Helsinki and all procedures involving human subjects were approved by the ethics committee of the Bavarian Chamber of Physicians in Munich. Written informed consent was obtained from all subjects.

### Assessment of glucose tolerance status

Prevalent diabetes was defined by either current use of a glucose-lowering medication or a self-reported diagnosis of T2DM, both confirmed by the individual's physician. The remaining participants without a known diabetes diagnosis underwent a standard oral glucose tolerance test (OGTT) to classify them by glucose tolerance status according to the 2003 American Diabetes Association diagnostic criteria^(^^14^^)^. Further details have been described elsewhere^(^^13^^)^. Individuals with an OGTT value of ≥7·0 mmol/l fasting or ≥11·1 mmol/l 2-h glucose were classified with undetected diabetes mellitus (UDM), also known as screen-detected diabetes. Prediabetes was defined as having impaired fasting glucose (5·6–6·9 mmol/l fasting glucose), impaired glucose tolerance (7·8–11·0 mmol/l 2-h glucose) or the combination of both. Normal glucose tolerance (NGT) was considered a fasting glucose value of <5·6 mmol/l and a 2-h glucose OGTT value of <7·8 mmol/l.

### Dietary assessment

Dietary intake was assessed in KORA FF4 participants via repeated 24-h food lists (24HFL) and one FFQ. The 24HFL is a closed list of >300 food items used to evaluate which foods were consumed over the previous 24 h. A detailed description has been provided elsewhere^(^^15^^)^. The FFQ was based on the German multilingual European Food Propensity Questionnaire (EFPQ) and encompassed approximately 148 food items and determined which foods were consumed, how frequently and in what amount over the past 12 months^(^^16^^)^. The first 24HFL was administered at the test centre. Participants were encouraged to complete web-based questionnaires to prevent the submission of incomplete data. However, a paper questionnaire could be made available upon request. A total of 1602 participants completed one FFQ and at least one 24HFL. Of these participants, 652 (40·7 %) completed two 24HFL and 826 (51·6 %) completed three 24HFL.

Habitual dietary intake was then calculated for these 1602 participants using an approach based on the National Cancer Institute method, a validated method that has the goal of reducing the error associated with traditional dietary assessment tools. The National Cancer Institute method approximates habitual dietary intake based on both the probability of consuming a food on a given day and the usual portion size in which a food is consumed^(^^17^^,^^18^^)^. Following the idea of this two-step method, consumption probability was first estimated for each individual and each food item based on 24HFL data using logistic mixed models, which were adjusted for age, sex, BMI, physical activity, smoking, education and food consumption frequency, as evaluated by the FFQ. Because the 24HFL do not include consumption amounts, they were predicted based on data from 24-h diet recalls obtained from participants of the Bavarian Food Consumption Survey II (BVSII). The BVSII study was designed as a representative, population-based, cross-sectional study to characterise the dietary and lifestyle habits of Bavarians. Linear mixed models were used to calculate the consumption amount for a consumption day for each food item in the BVSII study. The models were adjusted for age, sex, BMI, physical activity, education and smoking. The β-estimates calculated for each food intake variable from the BVSII data were then used to predict the habitual consumption amount for each food item for each individual in the KORA FF4 study, based on his or her characteristics. Finally, the habitual dietary intake was calculated for each individual by multiplying the estimated intake probability for each food item by the estimated consumption amount. The dietary intake data were then categorised into sixteen food groups and twenty-one food subgroups according to the European Prospective Investigation into Cancer and Nutrition (EPIC)-Soft classification scheme^(^^19^^)^. Nutrient intakes were derived from habitual food intake estimates using the National Nutrient Database (Bundeslebensmittelschlüssel; BLS 3.02). Fruits (g/d), vegetables (g/d), potatoes (g/d), total meat (g/d), red meat (beef and pork, g/d), poultry (g/d), processed meat (g/d), eggs (g/d), total dairy products (g/d), milk (g/d), yogurt (g/d), cheese (g/d), coffee (g/d), fruit and vegetable juice (g/d) and sugar-sweetened beverages (SSB, g/d) are the food groups and subgroups which were used as the main exposure variables. Alcohol (g/d), total fibre (g/d) and insoluble fibre (g/d) are the nutrients that were chosen. Insoluble fibre was selected as an alternative to whole grain intake, which is not included in the EPIC-Soft classification system. These selections were made based on the findings of previous research that indicated either a positive or negative association with the consumption of these food items and the prevalence of T2DM^(^^8^^,^^20^^–^^29^^)^. All selected food intake variables, excluding alcohol, were then divided by their standard deviation in order to give risk estimates per 1 sd. In accordance with the nutrient reference values of the German Nutrition Society (Deutsche Gesellschaft für Ernährung), alcohol intake was categorised as non-consumer (<5 g/d for men, <2 g/d for women), moderate (≥5–<20 g/d for men and ≥2–<10 g/d for women) or heavy consumption (≥20 g/d for men and ≥10 g/d for women), in order to examine potential differences in association between moderate and heavy alcohol intake^(^^30^^)^. Non-consumers were classified as <2 g/d or <5 g/d rather than 0 g/d due to the method used for dietary intake estimation in this study, which does not allow for an intake of 0 g/d.

### Assessment of covariates

Potential covariates were selected from the existing literature on diet and diabetes^(^^20^^,^^24^^,^^31^^–^^33^^)^. The selected covariates were age (years), sex (male/female), BMI (kg/m^2^), waist circumference (cm), family history of diabetes (yes/no/do not know), physical activity (active ≥1 h per week/inactive), smoking (current/ex-/never smoker), hypertension (≥140/90 mmHg or antihypertensive medication, yes/no) and education. In accordance with the German education system, education was categorised as <10 years, 10–<13 years and ≥13 years for the analyses including all participants, but had to be condensed to <13 years and ≥13 years for the analyses stratified by sex due to low frequencies in the <10 years group. Vocational training is also taken into account in this variable. Waist circumference, blood pressure and BMI were measured at the study centre in standardised fashion by trained examiners. Education (years), family history of diabetes, physical activity and smoking status (regular/occasional/ex-/never smoker) were assessed during a face-to-face interview conducted by trained investigators. Further details have been outlined elsewhere^(^^13^^,^^34^^)^.

### Statistical analysis

Baseline characteristics were examined according to glucose tolerance status and sex. Characteristics were expressed as median and 25th and 75th percentiles or percentage and number. Significant differences between glucose tolerance status groups were evaluated using the Kruskal–Wallis test for continuous variables and the *χ*^2^ test for categorical variables. Fisher's exact test was used if frequencies were too low. Habitual dietary intake was also described according to glucose tolerance status and sex using the mean and standard deviation and median and 25th and 75th percentiles. Significant differences between glucose tolerance status groups were assessed using the Kruskal–Wallis test. To assess the associations between dietary intake and diabetes (NGT, prediabetes, UDM and prevalent T2DM), multinomial logistic regression was performed using the *mlogit()* function from the R package *mlogit*. Two multinomial logistic regression models were fitted for each dietary intake variable of interest with different sets of covariates: the crude model was adjusted for age, sex and energy intake; the fully adjusted model was additionally adjusted for BMI, waist circumference, family history of diabetes, physical activity, smoking, education and hypertension. All analyses were performed for the total sample of participants and stratified by sex. *P* values <0·05 were considered statistically significant. All analyses were performed using RStudio version 1.0.143 and R version 3.4.0.

## Results

Of the 1542 participants included in the analyses, 789 (51·2 %) had NGT, 545 (35·3 %) had prediabetes, sixty-five (4·2 %) had UDM and 143 (9·3 %) had prevalent diabetes. Table 1 displays the characteristics of these individuals according to sex and glucose tolerance status. There was a statistically significant difference between participants based on glucose tolerance status for all characteristics in both sexes. The participants with prediabetes, UDM and prevalent diabetes were older, less educated, had a higher BMI and waist circumference and were less active than individuals with NGT. Hypertension and a family history of diabetes were more prevalent in participants with prediabetes, UDM and prevalent diabetes than in individuals with NGT.

**Table 1.** Characteristics of the study population* by glucose tolerance status and sex (Medians and 25th and 75th percentiles for continuous variables; percentages and numbers for categorical variables)

	NGT		Prediabetes		UDM		Prevalent diabetes		
	Median/%	25 %, 75 %/*n*	Median/%	25 %, 75 %/*n*	Median/%	25 %, 75 %/*n*	Median/%	25 %, 75 %/*n*	*P*†
Sex (*n*)									
Men	292		333		36		91		<0·001
Women	497		212		29		52		
Age (years)									
Men	54	47, 64	62	53, 71	70	65, 76	72	66, 76	<0·001
Women	53	46, 62	63	57, 71	67	65, 77	68	63, 76	<0·001
Education (<13 years)									
Men	51·4	150	60·1	200	75·0	27	74·7	68	<0·001
Women	61·0	303	76·9	163	75·9	22	78·8	41	<0·001
BMI (kg/m^2^)									
Men	26·2	24·3, 28·6	28·1	25·7, 30·9	29·3	27·0, 32·4	29·6	26·5, 33·4	<0·001
Women	24·7	22·3, 27·6	28·5	25·3, 31·6	31·5	26·4, 34·5	31·4	28·6, 35·8	<0·001
Waist circumference (cm)									
Men	97	91, 104	103	97, 111	107	101, 113	108	101, 118	<0·001
Women	84	77, 94	95	87, 104	99	93, 110	105	95, 112	<0·001
Physical activity (active)									
Men	65·1	190	59·2	197	30·6	11	36·3	33	<0·001
Women	69·8	347	61·3	130	48·3	14	48·1	25	<0·001
Smoking (current smoker)‡									
Men	16·8	49	13·5	45	8·3	3	8·8	8	0·004
Women	15·1	75	13·2	28	0·0	0	5·8	3	0·005
Smoking (ex-smoker)‡									
Men	44·2	129	55·0	183	55·6	20	68·1	62	
Women	39·4	196	33·0	70	27·6	8	28·8	15	
Family history of diabetes (yes)‡									
Men	23·3	68	24·9	83	27·8	10	39·6	36	0·007
Women	24·1	120	35·4	75	31·0	9	36·5	19	<0·001
Hypertension (yes)									
Men	27·8	81	49·5	165	83·3	30	74·7	68	<0·001
Women	20·8	103	46·2	98	55·2	16	75·0	39	<0·001

NGT, normal glucose tolerance; UDM, undetected diabetes mellitus.

* *n* 1542 (men, *n* 752; women, *n* 790).

† *P* values were calculated using the Kruskal–Wallis test for continuous variables and the *χ*^2^ test for categorical variables.

‡ Fisher's exact test was used to calculate *P* values due to low frequencies.

Table 2 characterises the dietary intake of men and women in this study according to glucose tolerance status, as well as the mean and standard deviation for each food item for the total population. Potato, processed meat, dairy products, milk, energy and alcohol intake differed significantly according to glucose tolerance status in both men and women. Egg, coffee and juice consumption varied significantly according to glucose tolerance status in men; and vegetable, yogurt, total meat and red meat consumption differed significantly between groups in women.

**Table 2.** Habitual dietary intake* of the study population by glucose tolerance status and sex (Mean values and standard deviations for total population, medians and 25th and 75th percentiles for glucose tolerance status groups)

	Total population (*n* 1542)		NGT (*n* 789)		Prediabetes (*n* 545)		UDM (*n* 65)		Prevalent diabetes (*n* 143)		
	Mean	sd	Median	25 %, 75 %	Median	25 %, 75 %	Median	25 %, 75 %	Median	25 %, 75 %	*P*†
Sex (*n*)											
Men	752		292		333		36		91		
Women	790		497		212		29		52		
Food item											
Fruit (g/d)											
Men	151	80	137	69, 208	146	84, 208	148	89, 215	136	101, 214	0·33
Women	160	79	145	95, 204	148	103, 211	156	111, 206	165	106, 222	0·39
Vegetables (g/d)											
Men	156	47	153	123, 190	147	124, 182	144	121, 166	143	117, 172	0·24
Women	193	64	186	152, 229	175	139, 215	170	144, 192	173	141, 220	0·04
Potatoes (g/d)											
Men	66	23	56	47, 69	63	52, 80	63	52, 86	66	57, 86	<0·001
Women	55	20	49	39, 61	53	44, 70	61	48, 76	59	49, 73	<0·001
Meat (total) (g/d)											
Men	144	41	139	115, 162	138	116, 166	157	134, 176	141	120, 160	0·13
Women	90	25	84	71, 99	85	74, 101	92	72, 108	95	84, 116	<0·001
Red meat (g/d)											
Men	35	13	34	26, 40	31	27, 40	38	29, 45	31	27, 40	0·23
Women	22	7	19	16, 25	21	18, 26	19	18, 21	20	18, 25	0·01
Poultry (g/d)											
Men	16	8	14	11, 21	13	11, 19	12	11, 19	13	11, 19	0·06
Women	14	7	11	9, 17	11	9, 15	11	9, 18	11	9, 16	0·76
Processed meat (g/d)											
Men	65	30	56	42, 72	60	46, 79	75	53, 95	63	50, 85	<0·001
Women	35	16	29	23, 39	32	26, 41	36	26, 51	41	34, 52	<0·001
Eggs (g/d)											
Men	17	13	11	8, 17	13	9, 20	16	11, 23	16	11, 22	<0·001
Women	13	10	10	7, 15	10	8, 15	10	8, 19	10	8, 14	0·65
Total dairy products (g/d)											
Men	175	100	176	111, 253	139	99, 221	130	95, 184	125	95, 212	0·001
Women	217	106	213	151, 288	179	127, 248	177	105, 269	171	111, 216	<0·001
Milk (g/d)											
Men	79	75	66	20, 138	53	17, 103	41	26, 66	44	16, 84	0·001
Women	103	80	93	47, 157	77	38, 124	61	18, 135	68	24, 117	0·003
Yogurt (g/d)											
Men	40	43	22	12, 59	20	12, 51	24	12, 42	14	11, 47	0·40
Women	54	47	41	18, 80	34	16, 67	35	16, 80	29	16, 57	0·03
Cheese (g/d)											
Men	30	14	29	19, 38	27	19, 37	25	20, 33	29	18, 40	0·59
Women	29	13	27	18, 37	26	18, 37	29	20, 38	25	18, 35	0·80
Coffee (g/d)											
Men	409	141	445	385, 498	433	344, 493	476	408, 501	472	369, 511	0·05
Women	392	128	413	354, 465	434	361, 472	448	373, 467	426	355, 466	0·37
Fruit and vegetable juice (g/d)											
Men	100	106	59	30, 166	50	28, 141	27	23, 95	30	24, 85	<0·001
Women	77	82	44	22, 104	36	20, 80	42	21, 108	29	19, 103	0·11
SSB (g/d)											
Men	75	165	11	6, 46	10	5, 35	9	5, 93	8	5, 57	0·46
Women	31	78	4	3, 8	4	3, 14	3	2, 4	4	3, 6	0·25
Nutrient											
Energy intake (kJ/d)											
Men	8892	1480	8983	8076, 9983	8722	7888, 9777	8713	7761, 9584	8443	7584, 9034	0·005
Women	6783	1188	6753	6059, 7578	6514	5885, 7301	6535	5990, 7580	6295	5759, 7262	0·006
Alcohol (g/d)											
Men	16	11	12	5, 23	16	6, 26	13	7, 19	9	4, 22	0·006
Women	5	5	3	2, 6	2	2, 5	2	1, 3	2	1, 4	<0·001
Fibre (total) (g/d)											
Men	18	5	18	15, 22	18	15, 21	17	15, 20	18	15, 20	0·44
Women	17	5	17	14, 20	16	14, 19	17	14, 19	16	14, 18	0·14
Insoluble fibre (g/d)											
Men	12	4	12	10, 15	12	10, 14	12	10, 13	12	10, 14	0·40
Women	12	4	11	9, 14	11	9, 13	11	9, 13	11	9, 12	0·13

NGT, normal glucose tolerance; UDM, undetected diabetes mellitus; SSB, sugar-sweetened beverages.

* Habitual dietary intake was calculated using a two-step method that combines estimated consumption amount and frequency.

† *P* values were calculated between glucose tolerance status groups using the Kruskal–Wallis test.

Table 3 shows the results of the crude multinomial logistic regression models. In the total study sample, fruit, vegetables, total meat, red meat, processed meat, total dairy products, yogurt, coffee, SSB, moderate alcohol, total fibre and insoluble fibre had statistically significant associations with glucose tolerance status. When stratified by sex, there was a significant association for total meat, red meat, processed meat, SSB, total fibre and insoluble fibre for both men and women. Coffee and heavy alcohol remained significant only for men, while yogurt and moderate alcohol remained significant only for women. The results of the fully adjusted models are presented in Table 4. A significant association was present in the total sample population for fruit, total meat, processed meat, coffee, SSB and moderate and heavy alcohol. Fruit intake was not significantly associated with prediabetes, but did show a statistically significant inverse association with UDM and prevalent diabetes, with OR of 0·71 (95 % CI 0·51, 0·98) and 0·77 (95 % CI 0·60, 0·98) per 1 sd in comparison with NGT, respectively. This association was not present when stratified by sex. Total meat intake was not significantly associated with prediabetes, but showed a statistically significant association with UDM (OR 1·99; 95 % CI 1·31, 3·00) and prevalent diabetes (OR 1·59; 95 % CI 1·13, 2·24) per 1 sd in comparison with NGT. When stratified by sex, a significant association remained only for UDM in men (OR 1·97; 95 % CI 1·23, 3·14) and for prevalent diabetes in women (OR 1·54; 95 % CI 1·03, 2·29) in comparison with NGT. A significant association with UDM and prevalent diabetes was also present for processed meat, with OR per 1 sd of 1·92 (95 % CI 1·38, 2·67) and 1·61 (95 % CI 1·20, 2·15), respectively. After stratification for sex, processed meat was significantly associated with UDM in men (OR 2·03; 95 % CI 1·34, 3·07) and with prevalent diabetes in both men and women (OR 1·53, 95 % CI 1·06, 2·19; and OR 1·58, 95 % CI 1·12, 2·23, respectively). In addition, SSB intake was significantly positively associated with prevalent diabetes in the total population (OR 1·17; 95 % CI 1·04, 1·33) and in men (OR 1·35; 95 % CI 1·00, 1·82). Moderate alcohol consumption was positively associated with UDM in men (OR 2·91; 95 % CI 1·10, 7·71).

**Table 3.** Energy-, age- and sex-adjusted associations between the consumption of various foods and nutrients and glucose tolerance status† (Odds ratios and 95 % confidence intervals per 1 standard deviation)

		Prediabetes (*n* 545)		UDM (*n* 65)		Prevalent diabetes (*n* 143)	
	sd	OR	95 % CI	OR	95 % CI	OR	95 % CI
Sex (*n*)							
Men		333		36		91	
Women		212		29		52	
Food item							
Fruit	80	0·91	0·80, 1·04	0·72*	0·52, 0·98	0·81	0·64, 1·01
Men	80	0·89	0·75, 1·07	0·75	0·50, 1·15	0·74	0·55, 1·01
Women	79	0·95	0·78, 1·16	0·62	0·37, 1·03	0·89	0·61, 1·28
Vegetables	59	0·92	0·80, 1·05	0·65*	0·44, 0·95	0·98	0·77, 1·26
Men	47	0·89	0·75, 1·06	0·70	0·45, 1·10	0·80	0·59, 1·09
Women	64	0·97	0·79, 1·18	0·58	0·32, 1·03	1·20	0·85, 1·70
Potatoes	22	1·05	0·60, 1·82	0·70	0·23, 2·17	1·21	0·54, 2·72
Men	23	1·43	0·67, 3·05	0·70	1·51, 3·23	1·53	0·54, 4·33
Women	20	0·71	0·31, 1·65	0·76	0·14, 4·10	0·92	0·24, 3·51
Meat (total)	43	1·57*	1·32, 1·88	3·14*	2·26, 4·37	2·89*	2·20, 3·80
Men	41	1·35*	1·10, 1·65	2·67*	1·86, 3·85	2·21*	1·64, 2·98
Women	25	1·46*	1·21, 1·76	2·17*	1·49, 3·16	2·65*	1·96, 3·58
Red meat	12	1·15	0·996, 1·33	1·43*	1·06, 1·92	1·28*	1·01, 1·63
Men	13	1·03	0·87, 1·22	1·47*	1·05, 2·04	1·20	0·92, 1·58
Women	7	1·30*	1·10, 1·54	1·09	0·71, 1·67	1·29	0·94, 1·76
Poultry	8	0·99	0·88, 1·12	1·11	0·85, 1·47	1·14	0·93, 1·40
Men	8	0·98	0·83, 1·16	1·06	0·72, 1·55	1·15	0·89, 1·50
Women	7	1·00	0·84, 1·21	1·19	0·80, 1·75	1·12	0·81, 1·55
Processed meat	28	1·54*	1·30, 1·82	2·80*	2·12, 3·70	2·60*	2·05, 3·31
Men	30	1·44*	1·17, 1·77	2·68*	1·91, 3·78	2·25*	1·68, 3·02
Women	16	1·37*	1·13, 1·66	2·09*	1·50, 2·91	2·43*	1·84, 3·20
Eggs	12	1·08	0·95, 1·22	1·11	0·88, 1·41	1·06	0·87, 1·28
Men	13	1·13	0·94, 1·36	1·10	0·78, 1·56	1·10	0·85, 1·44
Women	10	1·03	0·87, 1·22	1·15	0·84, 1·57	1·00	0·74, 1·35
Total dairy products	105	0·86*	0·75, 0·98	0·75	0·54, 1·04	0·90	0·72, 1·14
Men	100	0·88	0·74, 1·05	0·67	0·42, 1·07	0·94	0·71, 1·25
Women	106	0·84	0·68, 1·03	0·84	0·52, 1·35	0·83	0·57, 1·23
Milk	78	0·85	0·72, 1·00	0·73	0·48, 1·10	0·83	0·62, 1·11
Men	75	0·83	0·66, 1·03	0·62	0·35, 1·12	0·73	0·50, 1·06
Women	80	0·88	0·69, 1·12	0·84	0·48, 1·49	0·97	0·64, 1·48
Yogurt	45	0·87*	0·77, 0·99	0·95	0·71, 1·26	0·97	0·79, 1·21
Men	43	0·94	0·80, 1·12	1·03	0·72, 1·48	1·17	0·92, 1·49
Women	47	0·81*	0·66, 0·99	0·86	0·54, 1·36	0·67	0·44, 1·03
Cheese	13	0·95	0·59, 1·53	0·84	0·29, 2·45	1·71	0·79, 3·67
Men	14	0·74	0·40, 1·39	0·26	0·55, 1·21	1·47	0·56, 3·82
Women	13	1·34	0·65, 2·79	3·39	0·72, 16·00	1·65	0·44, 6·24
Coffee	134	0·88*	0·78, 0·99	0·98	0·75, 1·29	0·87	0·72, 1·05
Men	141	0·80*	0·67, 0·95	1·03	0·71, 1·49	0·90	0·70, 1·17
Women	128	0·98	0·82, 1·17	0·90	0·61, 1·33	0·77	0·58, 1·03
SSB	95	1·04	0·97, 1·13	1·19*	1·01, 1·39	1·24*	1·12, 1·39
Men	106	1·05	0·88, 1·24	1·41	0·98, 2·02	1·54*	1·19, 1·99
Women	82	1·10	0·93, 1·31	1·17	0·77, 1·80	1·37*	1·06, 1·76
Fruit and vegetable juice	130	1·01	0·89, 1·15	1·01	0·75, 1·36	1·02	0·82, 1·28
Men	165	0·98	0·83, 1·16	0·86	0·55, 1·33	0·87	0·63, 1·19
Women	78	1·03	0·86, 1·25	1·23	0·83, 1·82	1·30	0·94, 1·79
Nutrient							
Moderate alcohol‡	10	0·79	0·60, 1·05	0·73	0·40, 1·32	0·57*	0·36, 0·89
Men	11	1·05	0·68, 1·62	1·95	0·78, 4·91	0·91	0·48, 1·71
Women	5	0·70	0·49, 1·01	0·31*	0·13, 0·75	0·40*	0·21, 0·77
Heavy alcohol‡	10	1·22	0·87, 1·72	0·67	0·30, 1·52	0·72	0·41, 1·27
Men	11	1·65*	1·05, 2·59	1·14	0·38, 3·38	1·11	0·56, 2·20
Women	5	0·86	0·47, 1·57	0·86	0·23, 3·26	0·34	0·08, 1·55
Fibre (total)	5	0·74*	0·63, 0·88	0·42*	0·28, 0·66	0·64*	0·47, 0·87
Men	5	0·75*	0·60, 0·93	0·41*	0·23, 0·74	0·58*	0·39, 0·87
Women	5	0·76*	0·58, 0·99	0·39*	0·20, 0·78	0·72	0·43, 1·21
Insoluble fibre	4	0·74*	0·63, 0·87	0·42*	0·27, 0·65	0·65*	0·48, 0·88
Men	4	0·74*	0·59, 0·92	0·41*	0·23, 0·73	0·61*	0·42, 0·90
Women	4	0·77*	0·59, 0·99	0·39*	0·20, 0·77	0·69	0·41, 1·15

UDM, undetected diabetes mellitus; SSB, sugar-sweetened beverages.

* Significant result (*P* < 0·05).

† Models were adjusted for age and energy intake; additionally, models were either adjusted for sex or stratified by sex. Normal glucose tolerance status is the reference group. Habitual dietary intake was calculated using a two-step method that combines estimated consumption amount and frequency.

‡ Moderate considered ≥5 g/d–<20 g/d for men, ≥2 g/d–<10 g/d for women; heavy considered ≥20 g/d for men, ≥10 g/d for women.

**Table 4.** Fully adjusted associations between the consumption of various foods and nutrients and glucose tolerance status† (Odds ratios and 95 % confidence intervals per 1 standard deviation)

		Prediabetes (*n* 545)		UDM (*n* 65)		Prevalent diabetes (*n* 143)	
	sd	OR	95 % CI	OR	95 % CI	OR	95 % CI
Sex (*n*)							
Men		333		36		91	
Women		212		29		52	
Food item							
Fruit	80	0·90	0·78, 1·03	0·71*	0·51, 0·98	0·77*	0·60, 0·98
Men	80	0·89	0·73, 1·08	0·72	0·47, 1·12	0·74	0·53, 1·02
Women	79	0·91	0·74, 1·12	0·61	0·36, 1·03	0·78	0·51, 1·18
Vegetables	59	1·01	0·86, 1·18	0·84	0·57, 1·25	1·30	0·99, 1·70
Men	47	0·98	0·81, 1·20	1·02	0·65, 1·59	1·17	0·83, 1·63
Women	64	1·07	0·85, 1·34	0·62	0·32, 1·17	1·47	0·98, 2·19
Potatoes	22	1·16	0·64, 2·09	0·76	0·23, 2·51	1·73	0·72, 4·21
Men	23	1·79	0·80, 4·00	1·08	0·22, 5·46	2·93	0·93, 9·27
Women	20	0·69	0·28, 1·70	0·77	0·13, 4·59	1·01	0·22, 4·67
Meat (total)	43	1·01	0·81, 1·26	1·99*	1·31, 3·00	1·59*	1·13, 2·24
Men	41	1·01	0·80, 1·28	1·97*	1·23, 3·14	1·40	0·96, 2·06
Women	25	0·96	0·76, 1·23	1·32	0·82, 2·10	1·54*	1·03, 2·29
Red meat	12	0·96	0·82, 1·12	1·10	0·79, 1·54	0·96	0·73, 1·27
Men	13	0·90	0·75, 1·09	1·14	0·78, 1·65	0·92	0·67, 1·26
Women	7	1·10	0·91, 1·33	0·86	0·53, 1·40	0·96	0·66, 1·40
Poultry	8	0·94	0·83, 1·07	1·06	0·79, 1·43	1·11	0·88, 1·38
Men	8	0·96	0·81, 1·14	1·07	0·71, 1·60	1·16	0·87, 1·55
Women	7	0·91	0·75, 1·11	1·05	0·69, 1·62	0·96	0·65, 1·42
Processed meat	28	1·09	0·90, 1·32	1·92*	1·38, 2·67	1·61*	1·20, 2·15
Men	30	1·15	0·91, 1·45	2·03*	1·34, 3·07	1·53*	1·06, 2·19
Women	16	0·92	0·72, 1·17	1·33	0·89, 1·98	1·58*	1·12, 2·23
Eggs	12	1·04	0·91, 1·18	1·05	0·81, 1·34	0·95	0·77, 1·18
Men	13	1·07	0·88, 1·29	1·06	0·73, 1·55	1·00	0·75, 1·34
Women	10	0·99	0·82, 1·19	1·05	0·75, 1·47	0·88	0·63, 1·24
Total dairy products	105	0·88	0·77, 1·02	0·78	0·56, 1·09	0·94	0·73, 1·20
Men	100	0·92	0·77, 1·11	0·75	0·47, 1·19	1·02	0·75, 1·37
Women	106	0·87	0·70, 1·08	0·83	0·51, 1·34	0·77	0·50, 1·17
Milk	78	0·87	0·73, 1·04	0·73	0·47, 1·11	0·85	0·62, 1·16
Men	75	0·89	0·71, 1·12	0·71	0·39, 1·29	0·79	0·53, 1·19
Women	80	0·86	0·66, 1·12	0·76	0·42, 1·37	0·84	0·52, 1·34
Yogurt	45	0·88	0·77, 1·01	0·94	0·71, 1·26	0·96	0·77, 1·21
Men	43	0·92	0·77, 1·10	0·97	0·67, 1·39	1·12	0·87, 1·45
Women	47	0·85	0·69, 1·05	0·91	0·56, 1·49	0·64	0·40, 1·04
Cheese	13	0·97	0·58, 1·61	1·02	0·33, 3·15	2·02	0·87, 4·69
Men	14	0·68	0·35, 1·33	0·29	0·06, 1·49	1·92	0·64, 5·74
Women	13	1·65	0·74, 3·67	4·14	0·83, 20·76	2·07	0·47, 9·04
Coffee	134	0·86*	0·76, 0·98	0·98	0·74, 1·30	0·85	0·69, 1·05
Men	141	0·80*	0·67, 0·96	1·01	0·68, 1·49	0·84	0·63, 1·11
Women	128	0·94	0·77, 1·14	0·90	0·60, 1·36	0·80	0·57, 1·12
SSB	95	1·00	0·92, 1·08	1·11	0·93, 1·32	1·17*	1·04, 1·33
Men	106	0·96	0·79, 1·16	1·17	0·78, 1·76	1·35*	1·00, 1·82
Women	82	1·05	0·86, 1·28	1·10	0·67, 1·80	1·33	0·97, 1·82
Fruit and vegetable juice	130	0·97	0·85, 1·10	0·94	0·69, 1·26	0·92	0·72, 1·17
Men	165	0·97	0·81, 1·16	0·82	0·52, 1·27	0·82	0·59, 1·15
Women	78	0·96	0·78, 1·16	1·08	0·72, 1·63	1·08	0·75, 1·54
Nutrient							
Moderate alcohol‡	10	1·16	0·85, 1·57	1·36	0·72, 2·57	1·05	0·64, 1·71
Men	11	1·22	0·77, 1·93	2·91*	1·10, 7·71	1·30	0·65, 2·60
Women	5	1·22	0·80, 1·85	0·59	0·22, 1·55	0·96	0·45, 2·03
Heavy alcohol‡	10	1·78*	1·23, 2·59	1·22	0·52, 2·86	1·26	0·67, 2·36
Men	11	1·84*	1·14, 2·95	1·52	0·49, 4·72	1·45	0·69, 3·04
Women	5	1·81	0·91, 3·62	1·71	0·41, 7·16	0·77	0·15, 4·06
Fibre (total)	5	0·91	0·75, 1·11	0·64	0·40, 1·04	1·02	0·72, 1·44
Men	5	0·91	0·70, 1·17	0·66	0·35, 1·24	0·96	0·61, 1·50
Women	5	0·95	0·69, 1·29	0·55	0·25, 1·22	1·22	0·67, 2·23
Insoluble fibre	4	0·90	0·75, 1·09	0·63	0·40, 1·01	1·03	0·74, 1·45
Men	4	0·89	0·69, 1·14	0·64	0·34, 1·20	0·98	0·63, 1·52
Women	4	0·95	0·71, 1·29	0·54	0·24, 1·18	1·21	0·67, 2·19

UDM, undetected diabetes mellitus; SSB, sugar-sweetened beverages.

* Significant result (*P* < 0·05).

† Models were adjusted for age, energy intake, BMI, waist circumference, family history of diabetes, physical activity, smoking, education level and hypertension; additionally, models were either adjusted for sex or stratified by sex. Normal glucose tolerance status is the reference group. Habitual dietary intake was calculated using a two-step method that combines estimated consumption amount and frequency.

‡ Moderate considered ≥5 g/d – <20 g/d for men, ≥2 g/d – <10 g/d for women; heavy considered ≥20 g/d for men, ≥ 10 g/d for women.

Concerning diet and prediabetes, coffee consumption was significantly inversely associated with prediabetes (OR 0·86; 95 % CI 0·76, 0·98) per 1 sd in comparison with NGT. A significant association remained only in men once the analysis was stratified by sex (OR 0·80; 95 % CI 0·67, 0·96). Heavy alcohol intake was positively associated with prediabetes (OR 1·78; 95 % CI 1·23, 2·59), but this association remained statistically significant only for men after stratification by sex (OR 1·84; 95 % CI 1·14, 2·95). While total fibre, insoluble fibre, total dairy product and yogurt intake were inversely associated with prediabetes in the crude models, these associations were no longer statistically significant in the fully adjusted models (though risk estimates remained similar).

## Discussion

As a high percentage of individuals with prediabetes may progress to developing T2DM, a widespread disease with high personal and economic costs, it is imperative that interventions be focused not only on managing T2DM, but also on primary prevention^(^^1^^–^^3^^)^. Several studies have already demonstrated the effectiveness of diet and lifestyle interventions in slowing or preventing the progression of prediabetes to diabetes^(^^6^^,^^7^^)^. Additionally, the state of prediabetes may already be associated with negative outcomes^(^^5^^)^. Therefore, a full understanding of the interaction between diet and the development and progression of both prediabetes and diabetes is vital. A few studies have analysed the relationship between certain foods and the risk of prediabetes, but to our knowledge, the present study is the first to examine the associations between multiple food groups and prediabetes in a large sample^(^^8^^–^^12^^)^.

In the present study, fruit was inversely associated with UDM and prevalent diabetes, while total meat, processed meat, SSB and moderate alcohol were positively associated with UDM and/or prevalent diabetes. Coffee intake was inversely associated with prediabetes while heavy alcohol consumption was positively associated with prediabetes. These results are largely consistent with what is found in the existing literature^(^^11^^,^^12^^,^^20^^,^^23^^,^^35^^)^. In contrast to previous findings, red meat, vegetables, fibre (total and insoluble), fruit and vegetable juice, total dairy products, milk, yogurt, cheese, poultry, eggs and potatoes were not associated with glucose tolerance status in this population after adjustment for all covariates^(^^20^^,^^23^^,^^25^^,^^27^^–^^29^^,^^31^^,^^35^^–^^38^^)^. Overall, significant associations were seen more frequently in the total sample than when split by sex, probably because of the larger sample size, and more often in men than women.

Coffee intake was significantly inversely associated with prediabetes in the study population as a whole and in men when stratified by sex. A moderate, non-significant trend towards decreased risk was seen in most glucose tolerance categories. These results are consistent with a previous study by Lee *et al*.^(^^11^^)^, which found an inverse association between coffee intake and the risk of incident prediabetes and T2DM in individuals with known T2DM-related genetic mutations. Further studies have reported that coffee may lower the risk of developing T2DM or reduce the likelihood of progression from prediabetes to diabetes^(^^23^^,^^26^^,^^39^^,^^40^^)^. However, no previous study to our knowledge has identified an association between coffee consumption and prediabetes in a population-based sample.

Heavy alcohol intake was significantly positively associated with prediabetes in the total study population and in men, while a non-significant trend towards increased risk was present in all other groups except for women with prevalent diabetes. This is consistent with the results of one cohort study which found an increased risk of incident prediabetes and T2DM in men with heavy total alcohol intake, as well as two meta-analyses which found that heavy alcohol intake was associated with an increased risk of diabetes^(^^12^^,^^22^^,^^41^^)^. However, some studies have found that high alcohol intake had no effect on T2DM incidence^(^^42^^–^^44^^)^. In sensitivity analyses using 14 g/d (approximately 100 g per week) as the cut-off between moderate and heavy intake for both men and women, results were not significantly different^(^^45^^)^.

Several meta-analyses suggest that moderate alcohol intake, on the other hand, may be protective against prediabetes or T2DM^(^^41^^–^^44^^,^^46^^)^. However, moderate alcohol consumption was positively associated with UDM in men in our study. This is in accordance with a recent meta-analysis that found a modest increase in diabetes risk for men starting at a relatively low alcohol consumption level. In the same study, a protective effect of moderate alcohol intake was seen in women^(^^22^^)^. Cullman *et al*.^(^^12^^)^ also found that moderate alcohol consumption was protective in women, but not men. In our study, moderate alcohol was significantly inversely associated with prevalent diabetes and UDM in women in the crude analyses. Significance did not persist after full adjustment, but a non-significant inverse association did remain between moderate alcohol and UDM and prevalent diabetes in women. A potential reason why these results did not reach significance is that it was not possible to distinguish ‘former drinkers’ and ‘never drinkers’ from the reference category. As former drinkers are more likely to exhibit other health problems, therefore resulting in abstinence from alcohol, they would ideally be separated from the reference group^(^^47^^)^. Additionally, it should be noted that many studies have used cut-offs of between 30 and 60 g/d as the line between moderate and heavy alcohol intake. These levels are considerably higher than the cut-offs used in this study, which were chosen to align with actual alcohol consumption recommendations^(^^22^^,^^41^^,^^43^^,^^44^^,^^46^^)^.

In the crude analyses, total dairy products (total population) and yogurt (total population and in women) were significantly inversely associated with prediabetes. Although significance did not persist after full adjustment, a trend towards decreased risk of prediabetes remained for total dairy products, milk and yogurt in almost all categories. This is in line with a recent study that found that total dairy products, milk and yogurt were significantly associated with a decreased risk of incident prediabetes^(^^8^^)^. A number of meta-analyses have also found a significant inverse association between dairy products and the risk of diabetes^(^^33^^,^^37^^,^^48^^)^. However, results have been inconsistent in the literature, and we were not able to confirm an association between dairy product intake and glucose tolerance status after final adjustment for covariates.

The German Diabetes Risk Score, developed by the German Institute of Human Nutrition Potsdam-Rehbruecke (DIfE), considers age, height, high blood pressure, a family history of diabetes, waist circumference, physical activity, whole grain intake, meat intake, coffee consumption and smoking as major factors contributing to diabetes risk^(^^49^^)^. All of these risk factors were also taken into account in the present analyses. Older age, higher BMI, high blood pressure, positive family history of diabetes, larger waist circumference and inactivity all were more prevalent in individuals with prediabetes, UDM or prevalent diabetes than NGT. Smoking, however, was most prevalent in participants with NGT. Total meat and processed meat were significantly positively associated with UDM and prevalent diabetes, but red meat was not significantly associated with glucose tolerance status in this sample. A potential explanation for this discrepancy is the relatively low consumption of red meat in this population (Table 2)^(^^27^^,^^35^^)^. Insoluble fibre was taken as a potential proxy variable for whole grain intake, but while total fibre and insoluble fibre were significantly associated with reduced risk in nearly all glucose tolerance status categories in the crude analyses, neither insoluble nor total fibre was significantly associated with glucose tolerance status after adjusting for major potential covariates. These results are in accordance with a 2015 meta-analysis that found that intakes of total, cereal and vegetable fibre were significantly associated with a decreased risk of T2DM, but that the associations were no longer significant after adjustment for BMI^(^^24^^)^. Several other meta-analyses found that whole grain and/or dietary fibre intake to be associated with a lower risk of T2DM^(^^38^^,^^50^^,^^51^^)^.

Because of the cross-sectional nature of this study, these results cannot be interpreted as determining a causal relationship. Strengths of this study include a large sample size, which was initially randomly selected from the general population, and the large number of food items evaluated in this study. Other strengths include the use of an oral glucose tolerance test or physician-confirmed diagnosis to determine glucose tolerance status, the large number of covariates adjusted for, the consideration of sex-specific differences and the use of a sophisticated National Cancer Institute-based method for dietary intake evaluation^(^^17^^,^^18^^)^. In this study, participants with prediabetes, UDM or prevalent T2DM reported lower energy intake than participants with NGT, despite having a higher BMI (Table 2). This is an indicator that under-reporting of energy intake may be present, which could affect results. However, this may be partially explained by the higher physical activity of participants with NGT and the possibility that participants with prevalent T2DM were advised by their physicians to reduce their energy consumption in attempt to achieve a healthy weight. Such a change in dietary patterns could also have a resulting effect on the cross-sectional diet–diabetes association. However, we believe it is unlikely, as habitual dietary intake and the BMI of participants with prevalent T2DM and UDM (who would not have been advised to alter their diet) are very comparable. Still, this is one reason that confirmation with longitudinal studies is important. Selection bias as a result of the reduced size of the final study sample (due to lower participation in KORA FF4 than S4, as well as exclusion criteria in this analysis) could be another potential limitation. Lastly, our results have not been adjusted for multiple testing. The power would not have been sufficient to detect relevant associations in the fully adjusted analysis because of the relatively small number of participants in each group after stratification by glucose tolerance status and sex. However, when the conservative Bonferroni correction method is applied, those results with the smallest *P* values still remain statistically significant (total meat, processed meat and heavy alcohol; results not shown).

In conclusion, coffee consumption was significantly inversely associated with prediabetes, while heavy alcohol intake was significantly positively associated with prediabetes. Fruit consumption was inversely associated with UDM and prevalent T2DM. Alternatively, total meat, processed meat, SSB and moderate alcohol consumption were significantly positively associated with UDM and/or prevalent diabetes in this analysis. For the first time to our knowledge, we have examined associations between multiple food groups and prevalent prediabetes in a large sample. We have also identified, for the first time, a significant inverse association between coffee and prediabetes in a population-based sample. Additionally, consistent with a previous meta-analysis, our results suggest that moderate alcohol intake may be positively associated with T2DM in men, and we have replicated previous findings regarding a positive association between heavy alcohol consumption and prediabetes^(^^12^^,^^22^^)^. As dietary factors are modifiable, they are ideal targets for the primary prevention of T2DM. Our findings regarding coffee and heavy alcohol intake highlight potential targets for the primary prevention of prediabetes, in particular. These results should be taken into account in the planning of further studies, especially those experimental and prospective in nature, in order to further clarify these relationships.
